# Hippocampal Growth Factor and Myokine Cathepsin B Expression following Aerobic and Resistance Training in 3xTg-AD Mice

**DOI:** 10.1155/2020/5919501

**Published:** 2020-01-30

**Authors:** Gabriel S. Pena, Hector G. Paez, Trevor K. Johnson, Jessica L. Halle, Joseph P. Carzoli, Nishant P. Visavadiya, Michael C. Zourdos, Michael A. Whitehurst, Andy V. Khamoui

**Affiliations:** Department of Exercise Science and Health Promotion, Florida Atlantic University, Boca Raton, Florida 33431, USA

## Abstract

Aerobic training (AT) can support brain health in Alzheimer's disease (AD); however, the role of resistance training (RT) in AD is not well established. Aside from direct effects on the brain, exercise may also regulate brain function through secretion of muscle-derived myokines. *Aims*. This study examined the effects of AT and RT on hippocampal BDNF and IGF-1 signaling, *β*-amyloid expression, and myokine cathepsin B in the triple transgenic (3xTg-AD) model of AD. 3xTg-AD mice were assigned to one of the following groups: sedentary (Tg), aerobic trained (Tg+AT, 9 wks treadmill running), or resistance trained (Tg+RT, 9 wks weighted ladder climbing) (*n* = 10/group). Rotarod latency and strength were assessed pre- and posttraining. Hippocampus and skeletal muscle were collected after training and analyzed by high-resolution respirometry, ELISA, and immunoblotting. Tg+RT showed greater grip strength than Tg and Tg+AT at posttraining (*p* < 0.01). Only Tg+AT improved rotarod peak latency (*p* < 0.01). Hippocampal IGF-1 concentration was ~15% greater in Tg+AT and Tg+RT compared to Tg (*p* < 0.05); however, downstream signals of p-IGF-1R, p-Akt, p-MAPK, and p-GSK3*β* were not altered. Cathepsin B, hippocampal p-CREB and BDNF, and hippocampal mitochondrial respiration were not affected by AT or RT. *β*-Amyloid was ~30% lower in Tg+RT compared to Tg (*p* < 0.05). This data suggests that regular resistance training reduces *β*-amyloid in the hippocampus concurrent with increased concentrations of IGF-1. Both types of training offered distinct benefits, either by improving physical function or by modifying signals in the hippocampus. Therefore, inclusion of both training modalities may address central defects, as well as peripheral comorbidities in AD.

## 1. Introduction

Alzheimer's disease (AD) is the result of genetic susceptibility and environmental influences that present as an incurable progressive brain disorder affecting memory, decision-making, and vital neurobiological systems [1, 2]. AD disproportionately affects the elderly, and in 2016, more than 5 million Americans suffered from AD, with an estimated healthcare cost nearing $236 billion [3].

Neurotrophins derive from a highly conserved family of proteins in mammals essential for maintenance, survival, and neurogenesis [4]. In neurodegenerative diseases such as AD, critically important growth factors including brain-derived neurotrophic factor (BDNF) and insulin-like growth factor 1 (IGF-1) are downregulated, suggesting a pivotal role in the pathophysiology of AD [5–7]. Importantly, neurotrophin expression is upregulated in response to the bioenergetic challenge of voluntary exercise [8, 9]. As such, exercise may offer an alternative or adjuvant therapy to pharmacological interventions aimed at promoting neurotrophin expression [2, 5, 10] and possibly mitigating AD-related neurodegeneration [5, 11–14].

It is well documented that aerobic training (AT) can support brain health by delaying age-related cognitive decline and changing the trajectory of neurodegenerative diseases [5, 11]. For example, murine models of AD show that AT can increase levels of hippocampal BDNF and its high-affinity receptor TrkB concurrent with improved learning behaviors [11]. Similarly, human longitudinal studies have shown that AT helps to maintain and increase grey and white matter, while improving memory and executive function in healthy individuals and older adults with AD [15, 16].

Although studied far less than AT, resistance exercise has been shown to activate anabolic and neurotrophic signals. For instance, acute resistance exercise increased BDNF expression in older adults [17]. However, others reported no change in BDNF expression following either acute loading or chronic resistance training (RT) [18, 19]. Despite the conflicting results, RT may still modulate neuroprotection via IGF-1 [20]. Serum IGF-1 has been shown to increase in response to acute resistance loading and chronic RT, and this may support hippocampal neurogenesis [21, 22], whereas blocking brain uptake of IGF-1 eliminates these effects [23]. Finally, IGF-1 has been linked to AD pathophysiology due to its role in *β*-amyloid plaque clearance through the upregulation of essential proteins [12, 21, 24]. How RT affects hippocampal expression of BDNF, IGF-1, and *β*-amyloid in AD is not well defined at present.

In addition to direct effects on the brain, exercise has been suggested to regulate brain function through secretion of skeletal muscle-derived factors termed myokines. Recently, myokine cathepsin B (CatB) has been identified as a peripheral secretory factor that may mediate exercise's effects on brain health [25]. These findings were confirmed in rodents when CatB knockout mice failed to enhance adult hippocampal neurogenesis and spatial memory function in response to voluntary wheel running, suggesting a central role of CatB in aerobic exercise-induced improvements in cognitive function. CatB may mediate improvements in cognitive function by increasing levels of BDNF and degrading A*β* [25–28].

Resistance training (RT), which generates intermittent high muscular tension rather than sustained low muscular tension characteristic of AT, has been shown to increase CatB expression in healthy muscle [29]. To our knowledge, the relationship between CatB, RT, and AD has not been investigated. Therefore, the purpose of this study was to examine the effects of AT and RT on hippocampal BDNF and IGF-1 signaling, expression of *β*-amyloid, and myokine cathepsin B. *In situ* mitochondrial respiration and motor function were also measured as additional indices to assess the efficacy of exercise training.

## 2. Methods

### 2.1. Animals and Design

Three-month-old 3xTg-AD (*N* = 30) females were purchased from The Jackson Laboratory (Bar Harbor, ME). 3xTg-AD mice show AD-related pathology such as intracellular A*β* deposition and reduced performance in behavioral tests as early as 3 months of age [30, 31]. Continued intracellular A*β* accumulation and cognitive deficits occur at 6 months [30, 31], followed by extracellular A*β* deposits at ≥12 months [32]. Therefore, exercise training occurred during anticipated A*β* accumulation, and prior to the onset of A*β* plaque, similar to previous work on exercise and AD mice [33]. Mice were provided a three-day acclimation and not handled during this period. After acclimation, 3xTg-AD mice were randomly assigned to one of the following groups: sedentary (Tg, *n* = 10), aerobic training (Tg+AT, *n* = 10), or resistance training (Tg+RT, *n* = 10). All mice underwent pretraining assessments to obtain baseline values of physical function. Mice assigned to training groups then underwent a familiarization period for one week where they were introduced to their respective exercises. After familiarization, Tg+AT and Tg+RT performed their respective training for 9 weeks. At posttraining, the same assessments were repeated, followed by euthanasia and tissue collection. Mice were group housed, provided food and water *ad libitum*, and maintained on a 12 : 12 hr light : dark cycle. All experimental procedures were conducted with prior approval obtained from the Institutional Animal Care and Use Committee at Florida Atlantic University (protocol # A17-08).

### 2.2. Aerobic Training

Aerobic training was conducted on a 5-lane mouse treadmill (Harvard Apparatus). Mice went through a one-week familiarization period in which they ran at a speed of 15 m/min for 10 minutes daily on three nonconsecutive days, with the duration increased to 15 minutes by the third day of training. After the familiarization period, mice trained at a frequency of 5 days per week on the first week of training, gradually increasing from 30 minutes on day 1 to 60 minutes by day 5. Afterwards, the speed and frequency of training remained constant, but the length of training session was increased to 75 minutes on week 5 and 90 minutes on week 7. Only gentle reinforcement was used in which the researcher lightly touched the hips.

### 2.3. Resistance Training

Weighted ladder climbing was used to simulate RT. The ladder had a height of 100 cm and 1.5 cm grids and was placed at an 85-degree angle to the ground. A one-week familiarization period was conducted in which mice climbed the ladder unweighted for 4 repetitions with one-minute rest between repetitions. After familiarization, mice underwent 3 training sessions per week on nonconsecutive days for 9 weeks. The initial resistance was 50% of body mass for 16 total repetitions per training session with one minute of rest between repetitions. Load was increased by 12.5% of body mass weekly. After reaching 100% of body mass, total repetitions performed per session were decreased to 10. No positive or negative stimulus was used as incentive for climbing.

### 2.4. Grip Strength

The mice were allowed to grip the device with their forelimbs while restrained at the base of the tail. The animal was then pulled back by its tail until it released its grip. The force generated while the animal attempted to maintain its grip was quantified in grams by a strain gauge (Harvard Apparatus). The average force of four trials was calculated for each mouse.

### 2.5. Rotarod

Mice were placed on a rotating tube with an initial speed of 4 rotations per minute (rpm). Once all mice were positioned on the rod (5 mice), the assessment began. The speed of the rotarod increased at a rate of 1 rpm every 8 seconds (up to 40 rpm maximum). When an animal fell off the rotating tube, the timer was deactivated and the time and rpm recorded. After 500 seconds, if the mouse was still active the test was terminated. A total of 4 trials were given with 15 minutes of recovery between trials. The maximum time spent on the rotarod (i.e., peak latency) was used for analysis.

### 2.6. Tissue Collection

Tissue was collected 48 hours after the final training bout to control for acute effects of exercise. Mice were euthanized by ketamine/xylazine overdose delivered i.p. at 300/30 mg/kg. Skeletal muscle, brain, and vital organs were carefully isolated and removed. The right half of the hippocampus was immediately placed into preservation buffer (BIOPS: 2.77 mM CaK_2_EGTA, 7.23 mM K_2_EGTA, 5.77 mM Na_2_ATP, 6.56 mM MgCl_2_·6H_2_O, 20 mM taurine, 15 mM Na_2_PCr, 20 mM imidazole, 0.5 mM DTT, and 50 mM MES hydrate) and stored on ice for mitochondrial respiration experiments. The remaining hippocampus was then homogenized, protein extracted, and stored at -80°C. Remaining tissues were snap frozen and stored at -80°C.

### 2.7. High-Resolution Respirometry

To prepare the hippocampus, duplicate samples ~6 mg each were gently blotted dry on filter paper and weighed before being placed into the respirometer chambers. Chemical permeabilization was performed by addition of saponin directly into the respirometer chambers in accordance with Herbst and Holloway [34]. Oxygen flux per tissue mass (pmol·s^−1^·mg^−1^) was recorded in real time at 37°C in the oxygen concentration range of 550-350 nmol/ml using high-resolution respirometry (Oxygraph-2 k, Oroboros Instruments, Innsbruck, AT). *In situ* respiration was assessed using a protocol adapted from Burtscher et al. [35].

### 2.8. Respiration Data Analysis

Oxygen flux for the different respiratory states were corrected by subtracting the residual oxygen consumption. Fluxes from each duplicate measurement were averaged for statistical analysis. To determine flux control ratios, which express respiratory control independent of mitochondrial content, tissue mass-specific oxygen fluxes from the SUIT protocol were divided by maximal electron transfer system capacity as the reference state [36]. The respiratory control ratio (RCR), an index of coupling efficiency of the OXPHOS system, was calculated in the complex I linked state [35].

### 2.9. ELISA

Hippocampal tissue was homogenized in NP-40 lysis buffer containing protease and phosphatase inhibitors. IGF-1 concentration was measured in the hippocampal homogenate using IGF-1 mouse/rat ELISA kit per manufacturer guidelines (cat# MG100, R&D Systems, Minneapolis, MN).

### 2.10. Western Blotting

Protein was isolated from the hippocampus using the NP-40 lysis buffer containing a protease/phosphatase inhibitor cocktail (Halt, Thermo Fisher Scientific, cat# 78425 and 78428). For the gastrocnemius muscle, protein was extracted using an ice-cold lysis buffer (150 mM NaCl, 10 mM HEPES, 1 mM EGTA, 0.1 mM MgCl_2_, and 1% Triton X-100, pH 7.4) containing a freshly made protease/phosphatase inhibitor cocktail (0.5x Sigma-Aldrich P2714, 100 *μ*M PMSF, 0.1 *μ*M okadaic acid, and 1 mM orthovanadate). Protein concentration was determined via Pierce BCA protein assay kit (Thermo Fisher Scientific, Waltham, MA). Equal amounts of protein (35 *μ*g/lane) were loaded and separated by SDS-PAGE using 4–20% Criterion™ TGX™ Precast Gels (cat# 5671095, Bio-Rad, Hercules, CA) and electrotransferred to PVDF membranes. The membranes were blocked in 6% nonfat dry milk or 5% bovine serum albumin (BSA: in case of phospho-specific antibody) for one hour at room temperature, and then incubated at 4°C overnight with the primary antibody of interest. The primary antibodies used in this study included BDNF (cat# ab108319) and Cathepsin B (Cat# ab58802) from Abcam Inc., Cambridge, MA; IGF-1R (cat# 05-656) and p-IGF-1R (cat# ABE332) from Millipore, Temecula, CA; *β*-Amyloid (cat# sc-28365), CREB (cat# sc-271), and p-CREB (cat# sc-81486) from Santa Cruz Biotechnology, Santa Cruz, CA; and Akt (cat# 4691), p-Akt, (cat# 4060), GSK3*β* (cat# 9315), p-GSK3*β* (cat# 9322), MAPK 42/44 (cat# 9102), p-MAPK 42/44 (cat# 9101), *α*-tubulin (cat# 3873), and *β*-actin (cat# 3700) from Cell Signaling Technology, Danvers, MA. For secondary antibodies, we used peroxidase-conjugated horse anti-mouse IgG (cat#7076) and goat anti-rabbit IgG (cat# 7074) from Cell Signaling Technology, Danvers, MA. The immunoreactive protein reaction was revealed using the SuperSignal™ West Pico PLUS Chemiluminescent Substrate (cat# PI34580, Thermo Fisher Scientific). The reactive bands were detected by a ChemiDoc™ XRS+ imaging system (Bio-Rad), and density was measured using the ImageJ software (NIH).

### 2.11. Statistical Analysis

All data are reported as mean ± SE. A 3 (groups) × 2 (timepoints) factorial ANOVA was used to evaluate differences in physical function (i.e., grip strength and rotarod). Differences in protein expression were determined by one-way ANOVA. Muscle wet mass was analyzed by an unpaired *t*-test. Follow-up testing was conducted with Tukey's HSD to localize significant interaction or main effects. Statistical significance was set at *p* ≤ 0.05.

## 3. Results

### 3.1. Phenotype of Aerobic- and Resistance-Trained 3xTg-AD Mice

**Figure 1:**
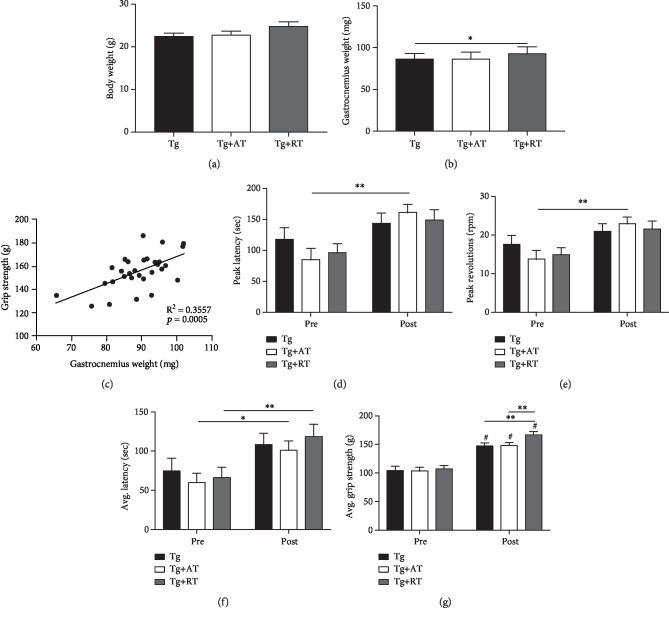
Phenotype of aerobic- and resistance-trained 3xTg-AD mice. Three-month-old 3xTg-AD mice were assigned to one of the following groups: nonexercised (Tg), aerobic trained (Tg+AT), or resistance trained (Tg+RT) (*n* = 10/group). Training was performed for 9 weeks, followed by tissue collection. Physical function was evaluated longitudinally, at pre- and posttraining. (a) Final body weight measured at the end of the experiment. (b) Wet weight of the gastrocnemius. (c) Association of gastrocnemius weight with grip strength. (d) Peak latency (time to fall in seconds) on the rotarod test. (e) Peak revolutions per minute (RPM) achieved on the rotarod test. (f) Average latency (time to fall in seconds) averaged across four trials on the rotarod. (g) Average forelimb grip strength across four trials. Data presented as mean ± SE. Differences determined by one-way ANOVA. ^∗^*p* < 0.05 and ^∗∗^*p* < 0.01.

No differences were observed for body weight (*p* > 0.05) (Figure 1(a)). Gastrocnemius mass was greater in Tg+RT compared to Tg (*p* < 0.05) (Figure 1(b)), consistent with resistance training-induced muscle hypertrophy. Gastrocnemius mass related linearly with grip strength (*r* = 0.59, *R*^2^ = 0.35, *p* < 0.05) (Figure 1(c)). Peak latency and revolutions were not different between groups at pretraining (*p* > 0.05) (Figures 1(d)–1(e)). Only Tg+AT significantly increased peak latency (+88%) and revolutions (+66%) from pre- to posttraining (*p* < 0.01) (Figures 1(d)–1(e)). Average latency was not different between groups at pretraining (*p* < 0.05) (Figure 1(f)). However, average latency increased (*p* < 0.05) from pre- to posttraining in Tg+AT (+68%, *p* < 0.05) and Tg+RT (+78%, *p* < 0.01) (Figure 1(g)). There were no differences in strength at pretraining (*p* > 0.05) (Figure 1(g)). All groups increased strength from pre- to posttraining; however, Tg+RT had significantly greater strength than Tg and Tg+AT at posttraining (+13% vs. both groups, *p* < 0.01) (Figure 1(g)), indicating greater improvement with resistance training.

### 3.2. Myokine Cathepsin B Response to Aerobic and Resistance Training in 3xTg-AD Mice

**Figure 2:**
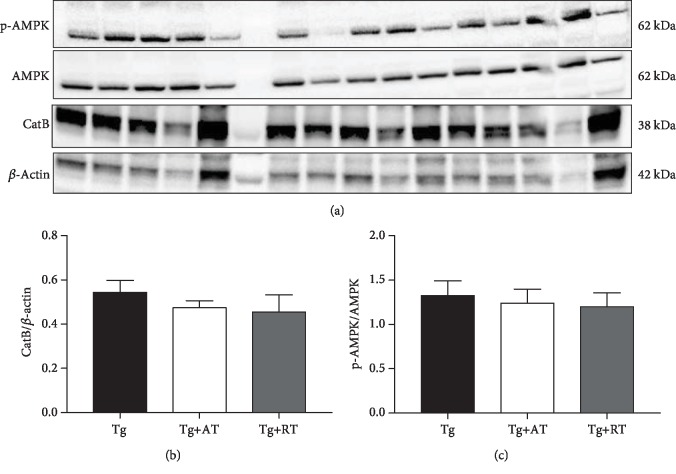
Unaltered AMPK activation and cathepsin B (CatB) expression in skeletal muscle of exercise-trained 3xTg-AD mice. (a) Immunoblots for CatB, p-AMPK, and AMPK in skeletal muscle homogenate. (b) CatB expression normalized to *β*-actin. (c) p-AMPK expression normalized to total AMPK. Data presented as mean ± SE. Tissues assayed from the nonexercised group (Tg), the aerobic-trained group (Tg+AT), or the resistance-trained group (Tg+RT) (*n* = 10/group). Differences determined by one-way ANOVA.

Myokine cathepsin B is a skeletal muscle-secreted factor shown to be important for exercise-induced improvement in cognitive function. Cathepsin B expression in skeletal muscle is regulated in part by the activity of AMPK. No group differences were detected for p-AMPK or mature cathepsin B in skeletal muscle (*p* > 0.05) (Figures 2(a)-2(c)).

### 3.3. Effect of Aerobic and Resistance Training on Hippocampal Mitochondrial Respiration

**Figure 3:**
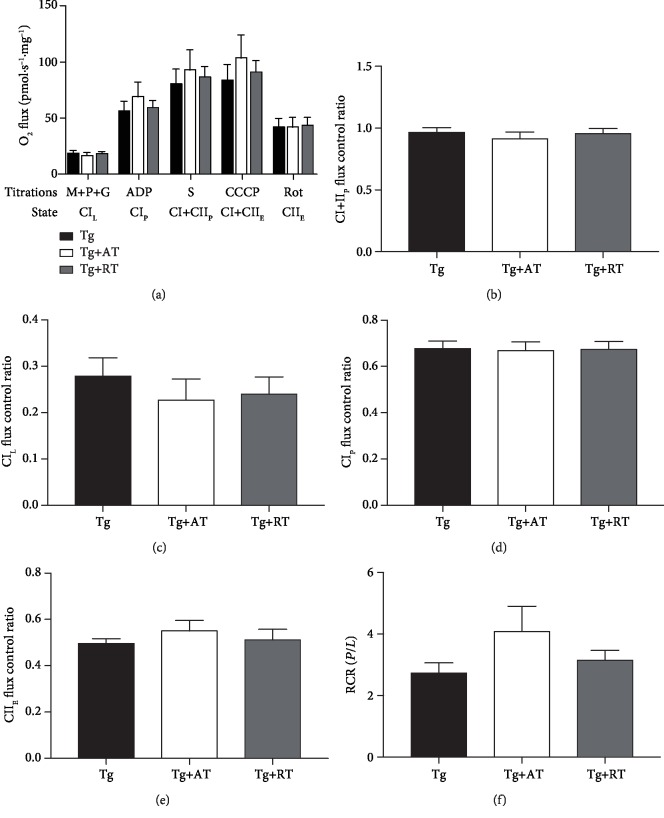
Unaffected mitochondrial respiration of the hippocampus in exercise-trained 3xTg-AD mice. (a) Mass-specific oxygen (O_2_) flux of the hippocampus determined *in situ* by a substrate-uncoupler-inhibitor titration protocol, including complex I-supported LEAK (CI_L_) through addition of malate, pyruvate, and glutamate (M+P+G); complex I-supported oxidative phosphorylation (OXPHOS) (CI_P_) by addition of adenosine diphosphate (ADP); complex I+II-supported OXPHOS (CI+II_P_) by addition of succinate (S); maximal electron transfer system (ETS) capacity (CI+II_E_) by stepwise titration of carbonyl cyanide m-chlorophenyl hydrazine (CCCP); and complex II ETS (CII_E_) by addition of rotenone (Rot). (b) Respiratory control ratio (RCR) determined by dividing CI_P_ by CI_L_. (c–f) Flux control ratios were calculated by normalizing mass-specific fluxes to maximal electron transfer system capacity (CI+II_E_). Shown are flux control ratios for (c) CI_L_, (d) CI_P_, (e) CI+II_P_, and (f) CI+II_E_. Data presented as mean ± SE. Tissues assayed from the nonexercised group (Tg), the aerobic-trained group (Tg+AT), or the resistance-trained group (Tg+RT) (*n* = 10/group). Differences determined by one-way ANOVA.

Mitochondrial dysfunction is associated with AD, and exercise may alter mitochondrial function. We therefore determined *in situ* mitochondrial respiration in saponin-permeabilized hippocampus by high-resolution respirometry. Tissue mass-specific fluxes were not different between groups (*p* > 0.05) (Figure 3(a)), suggesting no effect of training on hippocampal mitochondrial function per unit of tissue mass. RCR in the complex I-supported state, an index of OXPHOS coupling efficiency, was not different between groups (*p* > 0.05) (Figure 3(b)). Flux control ratios, which express respiratory control independent of mitochondrial mass, were determined as indicators of mitochondrial quality. No differences were observed in any of the flux control ratios calculated (*p* > 0.05) (Figures 3(c)-3(f)), implying no effect of training on hippocampal mitochondrial quality.

### 3.4. Hippocampal Neurotrophin and *β*-Amyloid Response to Aerobic and Resistance Training in 3xTg-AD Mice

**Figure 4:**
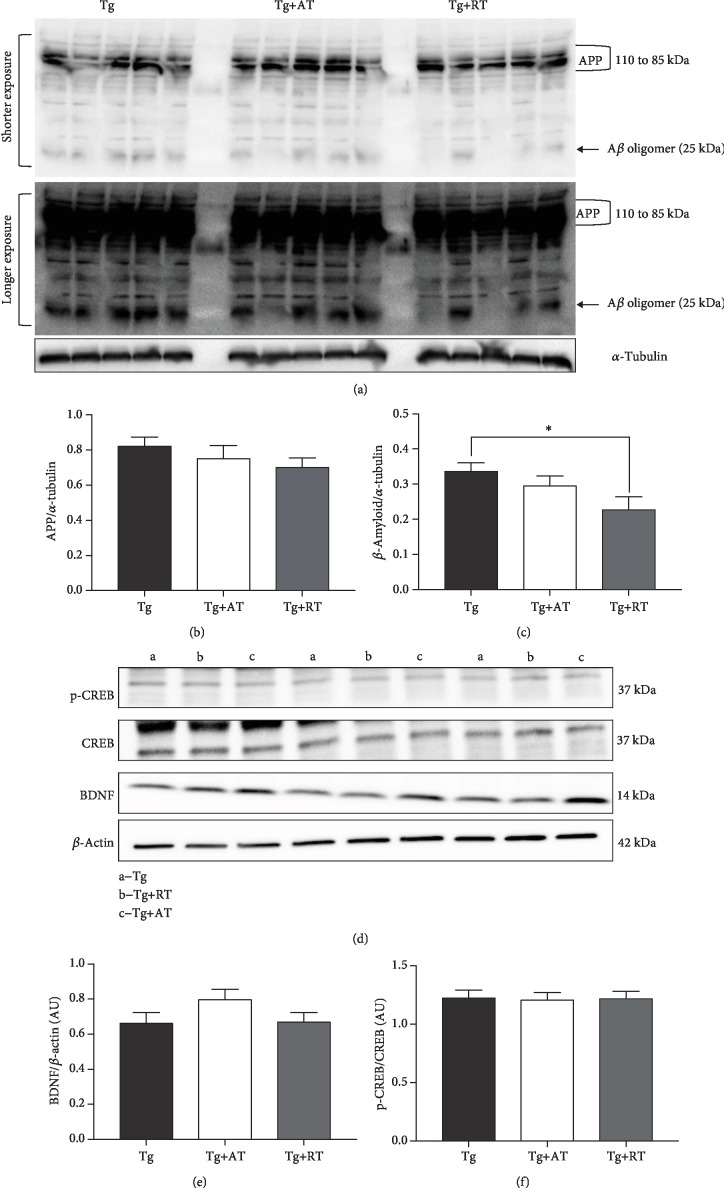
Reduced *β*-amyloid but unaltered CREB and BDNF in resistance-trained 3xTg-AD mice. (a) Representative immunoblots for amyloid precursor protein (APP) and *β*-amyloid probed in hippocampal homogenate. The shorter and longer exposure clearly revealed APP (110 to 85 kDa) and A*β* oligomer (25 kDa) bands, respectively. (b) Amyloid precursor protein (APP) normalized to tubulin. (c) *β*-Amyloid expression normalized to tubulin. (d) Representative immunoblots for CREB and BDNF expression. (e) p-CREB normalized to total CREB. (f) BDNF expression normalized to *β*-actin. Data presented as mean ± SE. Tissues assayed from the nonexercised group (Tg), the aerobic-trained group (Tg+AT), or the resistance-trained group (Tg+RT) (*n* = 10/group). Differences determined by one-way ANOVA. ^∗^*p* < 0.05 and ^∗∗^*p* < 0.01.

**Figure 5:**
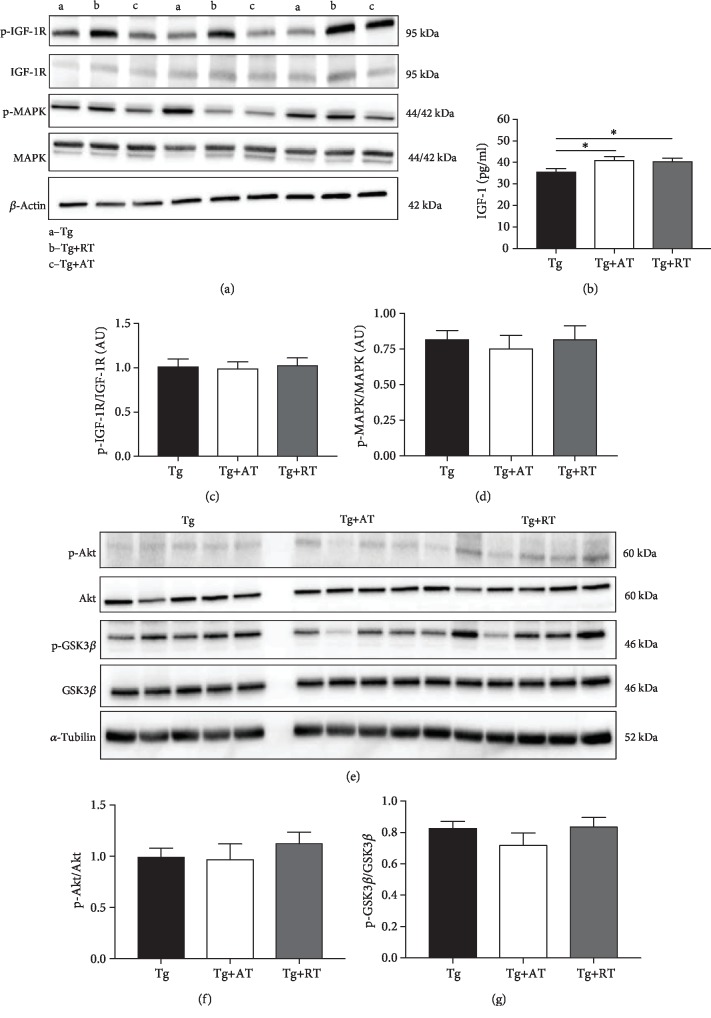
IGF-1 expression and signaling in the hippocampus of exercise-trained 3xTg-AD mice. (a) Representative immunoblots for IGF-1R and MAPK. (b) IGF-1 concentration in hippocampal homogenate measured by ELISA. (c) p-IGF-1R normalized to total IGF-1R. (d) p-MAPK normalized to MAPK. (e) Representative immunoblots for Akt and GSK3*β*. (f) p-Akt normalized to Akt. (g) p-GSK3*β* normalized to total GSK3*β*. Data presented as mean ± SE. Tissues assayed from the nonexercised group (Tg), the aerobic-trained group (Tg+AT), or the resistance-trained group (Tg+RT) (*n* = 10/group). Differences determined by one-way ANOVA. ^∗^*p* < 0.05 and ^∗∗^*p* < 0.01.

There were no differences in amyloid precursor protein expression (*p* > 0.05) (Figures 4(a) and 4(c)); however, *β*-amyloid was 32% lower in Tg+RT vs. Tg (*p* < 0.05) (Figures 4(b) and 4(c)). There were no differences in hippocampal p-CREB or BDNF (*p* > 0.05) (Figures 4(d)-4(f)). IGF-1 concentration in the hippocampus was significantly greater in Tg+AT (+15%) and Tg+RT (+13%) compared to Tg (*p* < 0.05) (Figure 5(b)). No differences were observed in the expression of p-IGF-1R, p-Akt, p-GSK3*β*, or p-MAPK (*p* > 0.05) (Figures 5(a) and 5(c)-5(g)).

## 4. Discussion

While the anabolic nature and associated benefits of RT are well established in the general population, very little is known regarding the effects of RT in AD. This study adds to the literature by contributing to a dialogue aimed at describing the role of RT in leveraging an adaptive brain as well as a peripheral response to bioenergetic demands. Moreover, as a clinical exercise modality, RT is unique in that it has the added benefit of being able to promote physical function and combat muscle wasting, comorbidities in AD.

We were somewhat surprised to find that RT significantly improved average latency on the rotarod. Perhaps the inherent instability of the near vertical weighted ladder climb triggered postural control mechanisms (e.g., vestibular and proprioception) as part of an adaptive motor response that included better dynamic balance. In addition, RT was superior to AT in terms of strength and muscle mass. Finally, and consistent with other studies, the low force protracted exercise bouts, hallmarks of AT and our protocol, translated to greater endurance and speed of movement (i.e., peak latency and RPM) when compared to RT.

While the current investigation did not observe a significant increase in the expression of BDNF within the hippocampus, previous literature suggests that gender differences may aid in the understanding of our results. Following a five-month aerobic exercise intervention, Venezia et al. saw BDNF gene expression to be significantly increased in both female and male wild-type mice, but BDNF protein to be only significantly higher in males and largely unchanged in females [37]. Along these lines, we note that BDNF protein was slightly greater in Tg+AT compared to Tg, and while not as robust as in mixed cohorts, is consistent with existing literature highlighting the use of aerobic exercise to upregulate this neurotrophin.

We note that both exercised groups increased hippocampal IGF-1 without changes in downstream signaling. One possible explanation is that our increased hippocampal IGF-1 is merely reflective of elevated circulating IGF-1. Previous work has shown that the concentration of hippocampal IGF-1 is highly related to circulating levels of IGF-1 [38]. Although we did not measure circulating IGF-1 because we did not collect blood, an increased circulating IGF-1 can be neuroprotective absent direct manipulation of the cellular cascades that regulate neuronal health. Outside of direct modulation of important cellular cascades such as those targeted in the present work, circulating IGF-1 levels have been implicated in the clearance of *β*-amyloid. In animal models, higher circulating IGF-1 levels have been associated with increased mobilization of clearing proteins—such as albumin and transthyretin—along with lower amyloid deposition and increased release of intracellular amyloid that may ultimately translate to decreased amyloid burden [39, 40]. Thus, exercise modulation of hippocampal and circulating IGF-1 may play other important ancillary roles in preventing accretion of *β*-amyloid oligomers.

Known to cross the blood-brain barrier from peripheral secretion in muscles during AT in humans, rhesus monkeys, and mice, cathepsin B (CatB) represents a potential therapeutic mechanism to combat AD. Specifically, CatB is a lysosomal cysteine protease that can lower AB levels [25]. However, in the present study, AT did not increase the expression of CatB in skeletal muscle. This may have been due to the use of involuntary treadmill training as our AT exercise. In a quintessential study [25], CatB expression in mouse muscle increased in response to voluntary wheel running, with distance ran reaching an average of over 19,000 meters per week. In the present study, animals ran an average distance of 4,500 meters in the first four weeks of training and 6,750 meters by the last week of training. Attempts to increase training volume on either exercise modality resulted in failure to complete the training session. Thus, it is possible that the total training volume in Tg+AT and Tg+RT may not have been sufficient to induce AMPK activation and CatB peripherally in skeletal muscle.

It is well known that mitochondrial dysfunction triggers AD pathology [39]. In the present study, we carried out mitochondrial function experiments using the hippocampus of 3xTg-AD mice following AT and RT. Our results indicated no significant alternations in any of the bioenergetics parameters. It is possible that the AT and RT protocols were not sufficient to stimulate improvements in hippocampal respiration, and may require greater intensity and/or prolonged training duration to see improvement in mitochondrial function in this mouse model. Alternatively, exercise-dependent mitochondrial adaptations may have been absent due to interference arising from the underlying disease.

A novel finding in our study was a reduction in hippocampal *β*-amyloid load in RT. Importantly, this finding closely parallels one of only several reports that *β*-amyloid levels were regulated by RT in AD. For example, Özbeyli et al. found a significant increase in hippocampal IGF-1 expression in conjunction with a decrease in *β*-amyloid following six weeks of ladder climbing in an AD rodent model [40]. Perhaps the reduction in *β*-amyloid load in RT represents a complementary adaptive response driven by the high force output and intermittent periods of rest. In support of this explanation, the low force and sustained nature of AT resulted in an increase in IGF-1 without a significant reduction in *β*-amyloid. Furthermore, it is conceivable that RT enhanced the expression of proteins, particularly those regulating *β*-amyloid clearance such as proteolytic degradation enzymes (e.g., *α*-secretase, neprilysin, and insulin-degrading enzyme), molecular chaperones (e.g., heat shock protein 70), and blood-brain barrier efflux proteins; however, these suggestions remain unverified and require further investigation. Thus, this finding suggests that regular RT may promote clearance of *β*-amyloid from the brain, a major pathological hallmark of AD. Finally, given the lower levels of *β*-amyloid associated with RT, a more comprehensive therapeutic strategy may be to combine RT and AT in an effort to maximize patient outcomes.

In summary, we found that *β*-amyloid burden was relieved by RT. This finding suggests that RT may be prophylactic in attenuating unwanted age-related and/or neurodegenerative changes in critical neural tissue/circuitry that underlie learning and memory, and ultimately vital biological systems. Given the propensity of AD to develop in the elderly, RT should be included as part of an exercise intervention prior to and during AD in order to increase strength, muscle mass, and function/independence, all comorbidities of AD.

## Data Availability

The data used to support the findings of this study are available from the corresponding authors upon request.
